# Change in EEG Activity is Associated with a Decrease in Tinnitus Awareness after rTMS

**Published:** 2021-05-17

**Authors:** G Carter, RB Govindan, G Brown, C Heimann, H Hayes, JC Thostenson, J Dornhoffer, T Brozoski, TA Kimbrell, A Hayar, B Shihabuddin, GA James, E Garcia-Rill, PR Padala, M Mennemeier

**Affiliations:** 1 Department of Anatomy and Physiology, National Park College, USA; 2 Prenatal Pediatrics Institute, Children’s National Hospital, USA; 3 Department of Neurobiology and Developmental Sciences, University of Arkansas for Medical Sciences USA; 4 Department of Biostatistics, University of Arkansas for Medical Sciences, USA; 5 Department of Otolaryngology, University of Arkansas for Medical Sciences, USA; 6 Division of Otolaryngology, Southern Illinois University School of Medicine, USA; 7 Section of Psychiatry, Central Arkansas Veterans Healthcare System, USA; 8 Department of Neurology, University of Arkansas for Medical Sciences, USA; 9 Department of Psychiatry, University of Arkansas for Medical Sciences, USA; 10 Central Arkansas Veterans Healthcare System, Geriatric Research Education and Clinical Center, USA; 11 Section of Neurology, Central Arkansas Healthcare System, USA

**Keywords:** tinnitus, repetitive transcranial magnetic stimulation, rTMS, 1Hz, 10Hz, placebo, thalamocortical dysrhythmia, TCD, EEG, spectral power, coherence

## Abstract

**Objective:**

To examine how 1Hz and 10Hz rTMS temporarily influence ratings of tinnitus loudness, annoyance, and awareness. The thalamocortical dysrhythmia (TCD) model of tinnitus was tested by examining changes in spectral power and coherence of resting state EEGs from baseline to each phase of treatment and correlating these data with change in tinnitus.

**Methods:**

Nineteen participants completed a double-blind, placebo (sham rTMS) controlled, within-subjects study with crossover between the two active rTMS treatment conditions. An imposed order effect, sham rTMS first, eliminated drift of active treatment into the placebo condition. The primary outcome measures were analogue ratings of tinnitus loudness, annoyance, and awareness, assessed repeatedly at baseline and during treatment, and 64 channel, resting state EEGs collected at baseline and the end of each treatment phase. Active rTMS consisted of 1800 pulses at 110% of motor threshold over temporal cortex delivered at 1Hz and 10Hz over four days. The research design also examined the effect of rTMS immediately following stimulation, regression to the mean in tinnitus ratings made over multiple days, and differences between treatment responders and non-responders.

**Results:**

There was no immediate effect of rTMS on tinnitus during a single rTMS session. Regression to the mean in tinnitus ratings occurred over three days of baseline and four days of treatment (both sham and active rTMS). After accounting for regression to the mean in the statistical model, 1Hz rTMS led to a significant decrease in tinnitus awareness from baseline and 10Hz rTMS trended in the same direction, whereas sham rTMS showed little change from baseline other than regression to the mean. Changes from baseline in spectral power of the resting state EEG provided partial support for predictions based on TCD model of tinnitus for active 1 and 10Hz rTMS but not sham rTMS. However, only an increase in beta coherence correlated significantly with a decrease in tinnitus awareness. Changes in the EEG were robust in treatment responders but absent among non-responders and during sham rTMS.

**Conclusions:**

A positive response to rTMS for tinnitus is associated with an rTMS-induced change in beta coherence of the EEG. Increased beta coherence may be a biomarker of the rTMS effect; a “top-down” modulation of the EEG that promotes habituation to tinnitus. Participants whose tinnitus did not improve after rTMS did not show any changes in the EEG.

## Introduction

Tinnitus, a phantom sound perception in the absence of acoustic simulation, is a common neurological disease that alters function in multiple brain systems [1]. Tinnitus is strongly associated with hearing loss but also occurs in people with normal audiograms. Tinnitus comorbidities include anxiety, depression, sleep disturbance, cognitive and social problems. Sixteen million people in the US report hearing tinnitus every day, 12 million seek treatment, and 3 million are unable to work. There is no cure. Behavioral interventions can help patients cope, but they do not typically reduce tinnitus perception [2].

Repetitive transcranial magnetic stimulation (rTMS) is a medical device that can reduce tinnitus perception [3]. During rTMS, a brief focused magnetic field, or pulse, is delivered over the scalp via a stimulation coil and each pulse creates a small electrical field within the brain. TMS pulses can be delivered repetitively and at different frequencies and intensities of stimulation [4]. Effects of rTMS can be observed locally beneath the stimulation coil and in distant, functionally connected brain regions in one or both hemispheres. Most rTMS studies of tinnitus deliver pulses over the temporal cortex but frontal lobe stimulation can also be effective [1]. Both temporal and frontal stimulation might be effective for tinnitus because rTMS can effect functionally connected brain regions.

Low (e.g., 1Hz) and high (e.g., 10Hz) frequencies of rTMS are thought to have different effects on cortical activation beneath the stimulation coil [5]; however, both frequencies have been shown to improve tinnitus [6]. An extensive review of 20 placebo-controlled studies for tinnitus, involving 601 patients, Lefaucheur et al. [3] found that a course of rTMS (e.g., 4-5 days) can reduce tinnitus perception in approximately 50% of patients and that the beneficial effect is temporary, typically lasting days to weeks. A positive response to rTMS was defined as a reduction of 30% or more on a visual analogue scale or a 5-point reduction or more on a self-report questionnaire. The same review, however, rated rTMS as having only “possible efficacy” for tinnitus because “the results of clinical trials are mixed due to methodological and practical problems that must be resolved before rTMS can be translated into a clinical application for tinnitus”.

The current study sought to investigate methodological uncertainties about how rTMS may reduce tinnitus. The study examined the timeframe over which rTMS alters tinnitus - immediately following stimulation versus cumulatively over several days. Understanding when rTMS influences tinnitus can address confusion over how low and high frequencies may have similar effects even though they should have different immediate effects on cortical activation. To do so, participants rated tinnitus using analogue scales before and after every rTMS treatment, in addition to the more common approach of using questionnaires to measure tinnitus at baseline, after a course of treatment, and at follow up.

The analogue ratings measured tinnitus loudness, annoyance, and awareness in daily life. Three separate ratings were used in order to determine if rTMS promotes habituation to tinnitus. In our previous rTMS studies, a number of subjects reported anecdotally that their tinnitus was “gone” after a few days of treatment. Yet, they rated tinnitus loudness and annoyance as similar to baseline, explaining that they simply did not notice tinnitus anymore in daily life. Behaviorally, habituation is defined as a decreased response to repeated internal or external sensory stimulation [7], such as failing to continue to notice a ticking clock in a room over time. Neurologically, habituation reflects a balance of dynamic neural activity involved in sensory gating. Ablation and electrophysiological studies in humans indicate that the prefrontal cortex gates sensory information from the thalamus via cortico-limbic-reticular-cortical circuits [8]. rTMS might reduce tinnitus perception by promoting habituation – reduced awareness of tinnitus but not necessarily a reduction in loudness or annoyance when focusing on tinnitus.

Applying rTMS as a treatment for tinnitus has inherent methodological challenges. As mentioned above, as many as half of patients with tinnitus may simply not respond to rTMS and there is no way of knowing in advance who will and who will not respond. Measuring tinnitus is also complicated. Merely asking a patient to rate tinnitus repeatedly can introduce measurement artifacts like regression to the mean – the tendency for initial ratings to regress toward a mean value upon subsequent ratings. Regression to the mean can mimic improvement or decline. Finally, placebo effects are common in rTMS studies [3] which necessitates the use of realistic sham rTMS condition – one that mimics the look, feel, and sound of active treatment [9]. To address these issues, the current study used an extended baseline and repeated assessments to measure regression to the mean, responders and non-responders were compared in follow up analyses, and a realistic sham rTMS treatment was used to examine placebo effects.

The current study used a double blind, within-subjects design with crossover between active treatments (1 and 10Hz rTMS) to investigate the temporary effects of a 4-5 day course of rTMS on tinnitus perception. TMS was delivered over the temporal cortex. An order effect was imposed to prevent carry-forward effects of active treatment into the sham condition. Possible mechanisms of the rTMS effect on tinnitus were investigated by testing the thalamocortical dysrhythmia (TCD) model of tinnitus through analysis of change in resting state EEGs obtained before and after each course of rTMS.

The TCD model [10,11] explains tinnitus as a process invlolving the loss of normal depolarizing sensory input from the cochlea to auditory nuclei of the thalamus. Deafferentation results in hyperpolarization that causes cells to switch to burst firing mode. The ionic mechanism is activation of the hyperpolarization-activated cation channels [12] and an increased expression of low threshold spikes (T-type calcium channels) [13]. At a circuit level, burst firing slows thalamocortical rhythms over the auditory cortex. It increases spectral power in the delta (~4Hz) to theta (~8Hz) frequency bands and it reduces power in the alpha (~10Hz) band. Both EEG and MEG recordings in patients with tinnitus reveal increased power (localized synchrony over temporal cortex) and increased coherence (global synchrony with other brain regions) in the delta-theta frequency bands, as well as reduced power and coherence in the alpha bands. Deafferentation also induces a second process thought to create phantom sound perception. Regions of neural contrast or “edge effects” are created by the failure of deafferented cell assemblies in auditory cortex to laterally inhibit the afferented cell assemblies. Edge effects produce synchronized activity that increases power in the gamma frequency band (>30Hz). Increased gamma power becomes “coupled” with increased power in the low frequency, delta-theta range. This coupling is a signature pattern associated with tinnitus [14]. By contrast, healthy normal subjects show increased power and coherence in the alpha range [10].

Spectral power and coherence of the EEG were analyzed to determine how rTMS alters TCD. Spectral power refers to localized synchrony of the EEG signal for a given frequency band (i.e., delta (1–3.5Hz), theta (4–8Hz), alpha (9–12Hz), beta (14–28Hz) and gamma (30–50Hz). Coherence refers to global synchronization between one electrode and all other electrodes. If high coherence exists, such as following active but not sham rTMS, then active rTMS may be viewed as promoting neural synchronization across electrodes for a given frequency band [15].

It was hypothesized that each TMS pulse would simultaneously activate the deafferented and afferented cell assemblies in temporal cortex. In fact, because corticothalamic projections out-number thalamocortical afferents by 10 to 1 [16], cortical stimulation may have an amplified effect on the thalamus which could change the expression of low threshold spikes. Accordingly, we predicted that active rTMS would shift spectral power over temporal cortex and coherence among network connections from the low-frequency EEG bands (delta-theta) to the higher-frequency bands (alpha) and that this change would correlate with tinnitus improvement. Repeating rTMS over 4-5 days may counter edge effects and promote homeostasis in temporal cortex and functionally connected brain regions. Reduced edge effects predict a reduction in gamma band power.

## Methods

### Participants

The participants in this report are a subset of a larger clinical trial that will be reported in its entirety in a subsequent manuscript. Participants in this report represent all those in the trial who had 64 channel EEG recordings. (The larger trial was subsequently modified to include a new 128 channel EEG system.) By the time of the last participant to receive 64 channel EEG, a total of 97 people had responded to study advertisement and were contacted. Twenty-four failed the initial screening, 30 could not commit to the required treatment schedule, and 2 declined due to a fear that tinnitus might worsen. All subjects enrolled in the study completed an informed consent process and signed a written informed consent form, consistent with ethical standards of the 1964 Declaration of Helesinki. The study was approved by the University’s Institutional Review Board (IRB). It operated under an IDE held by the study sponsor. The trial is registered in ClinicalTrials.gov (NCT00926237).

Forty-one participants were consented. Ten participants completed an early version of the protocol that did not include three baseline measures or ratings of tinnitus awareness and so these data were excluded from analysis. Five participants failed inclusion/exclusion criteria after consent. Seven withdrew before randomization. Two participants reported having a first-degree relative with epilepsy. They were allowed to enter under an exemption by the independent study monitor but could only receive rTMS at 1Hz. Nineteen participants were ultimately randomized to the treatment conditions.

#### Inclusion criteria

1) tinnitus symptoms for at least 6-months; 2) ability to comprehend study procedures and sign a written informed consent form; 3) pass the Transcranial Magnetic Stimulation Adult Safety Screen (TASS); 4) comply with the study and follow-up procedures; 5) agree to be videotaped during rTMS sessions for safety monitoring; 6) persons taking selective serotonin reuptake inhibitors (SSRIs) for tinnitus related depression must maintain a stable dose for 3 months without changes to their medication during their study participation; and 7) age between 18-89 years.

#### Exclusion criteria

1) history of epilepsy (or a first-degree relative diagnosed with epilepsy), head injury resulting in loss of consciousness for more than 10 minutes, aneurysm, stroke, previous cranial neurosurgery, acoustic neuroma, or glomus tumor; 2) active Meniere’s disease; 3) diagnosis of a neurological or major psychiatric disorder (excluding tinnitus related depression or anxiety); 4) having non-dental metal implants or shrapnel in the head or neck, a pacemaker, or other medical device implants that may interfere with the magnetic field; 5) pregnancy or the possibility of becoming pregnant, due to the lack of contraception use during the study; 6) being within 3 months of taking medications that lower seizure threshold or reduce cortical excitation (e.g., tricyclic antidepressants, benzodiazepines, bupropion, or anticonvulsants); 7) consuming alcohol within 72 hours prior to a rTMS session; 8) having profound hearing loss (≥90 dB at 4000Hz); or 9) being unable to complete English only study instructions or questionnaires.

### Measures

#### Tinnitus ratings

Verbal and visual analog scales that ranged from 0-100 were used to rate tinnitus loudness, annoyance, and awareness. For verbal analogue ratings (VARs) subjects were instructed to “rate tinnitus using a number between 0 & 100 with 0=“no tinnitus” and 100=“painful tinnitus”. Participants were instructed to rate tinnitus loudness, annoyance, and awareness in daily life separately for each ear. For visual analogue ratings, subjects were instructed to “place a mark on a line to rate your tinnitus” (i.e., the line mark (LM) rating). The line was 100 mm long and labeled “low” on the left end and “high” on the right end. Line mark ratings were converted to a number by measuring from the “low” end of the line to the subject’s mark and converting that distance to a percentage of the total line length.

#### Tinnitus comorbidities, questionnaires, and frequency and loudness matching

The Beck Depression Inventory (BDI) and the State Trait Anxiety Inventory (STAI) were used to assess comorbid depression and anxiety. Tinnitus questionnaires included the Tinnitus Handicap Inventory (THI) and the Tinnitus Handicap Questionnaire (THQ). Participants also completed the Tinnitus Assessment Package/Tinnitus Loudness Matching (TAP/TLM) computer program [17]. The TAP includes 4 questionnaires: THI, Hyperacusis Questionnaire (HQ), Tinnitus Experience Questionnaire (TEQ), and Tinnitus Interview Questionnaire (TINTQ). The TLM is a computer program that uses an amplifier (Natural Sound AV Receiver, Yamaha, Hamamatsu, Shizouka, Japan), a sound generator (Stanford Research Systems, Sunnyvale, CA, USA), and acoustic, noise cancelling headphones (BOSE, Framingham, MA, USA) to allow participants to match the perceived loudness of their tinnitus with real sounds. During the TLM participants match sounds generated by the program to the frequency and loudness of their perceived tinnitus. Sounds are presented in five frequency ranges (500Hz, 1kHz, 2kHz, 4kHz, and white noise) and in ascending and descending decibel levels. Two matching level scores are created: the hearing loudness (HL) and the sensation loudness (SL). The SL is a combination of HL and hearing threshold.

### Research design, study procedures and analysis

The study design was a double-blind, sham-controlled, within-subjects, clinical trial with participant crossover between the active 1Hz and 10Hz rTMS conditions. Participants were informed during the consent process that they would receive sham and active treatments but that they would not be told what they were receiving. All participants received sham rTMS first to prevent carry forward effects of active treatment into the sham condition. Participants were then randomized in permuted blocks by the biostatistician (JT) to the 1Hz and 10Hz active rTMS conditions. Participants crossed over to the second frequency of stimulation after completing the first. The two participants who entered on an exemption only received active rTMS at 1Hz. A three-day baseline preceded the sham treatment, and a 2-month assessment followed the end of treatment. A three-week washout with assessment at days 2, 9 and 16 separated each phase of treatment. Participants and study personnel who collected and analyzed data were blind to treatment condition (the person delivering treatment could not be blinded). The primary outcome measures were the VAR of tinnitus awareness, annoyance, and loudness and the measures of EEG spectral power and coherence. All other measures were secondary. EEG analysis focused on electrodes over temporal cortex (T7, T8, TP9, TP10, TP7, TP8, FT7, and FT8) both ipsi- and contralateral to rTMS. Effects of the intervention were assessed at multiple levels (i.e., before and after each rTMS session, on each day of treatment, by phase of treatment (baseline, sham and active 1 & 10Hz), during washout, and at follow up.

### Assessment schedule

All study measures were recorded at baseline, at the end of each treatment week, and at the 2-month study visit. In addition, the TAP/TLM assessment was completed at the beginning of each treatment week; both the VAR and LM ratings were measured immediately before and immediately after every rTMS session; the VAR was also measured over three days at baseline; and the VAR and the Tinnitus Handicap Questionnaire (THQ) were measured on days 2, 9, and 16 during the treatment week washout phases.

### Sham and active rTMS

rTMS was delivered using a Magstim® Super Rapid stimulator and visually identical, Magstim®, sham and active coils (i.e., Magstim®, air-film, figure-of eight, 70-mm coils: Magstim® Company, Whitland, Wales, UK). To mimic the sound of active TMS, sham stimulation was always set at 45% of the maximal stimulator output (MSO). To mimic the feel of active TMS, carbon rubber electrodes were placed on the scalp beneath the coil to generate scalp muscle twitching upon each pulse of sham rTMS [9]. A trigger output from the Magstim® stimulator activated a DS3 Stimulator (Digitimer Ltd., Welwyn Garden City, Hertfordshire, U.K) which sent an electrical pulse to the electrodes (between .9-15mV and 500-μsec in length). The active TMS coil was set at 110% of the daily motor threshold (MT). MT was determined by placing the active TMS coil over motor cortex and delivering pulses of increasing intensity to identify the smallest MSO needed to elicit a motor evoked potential (MEP) of at least 50 mV from the abductor polaris brevis of the contralateral hand (or a visual confirmation of a thumb twitch) on at least 3 out of 6 trials. Carbon rubber electrodes were also placed beneath the active rTMS coil but were not activated during stimulation.

Participants received three, 4-day courses of rTMS (i.e., sham rTMS delivered at either 1Hz or 10Hz and active rTMS delivered either at 1Hz first and then 10Hz or at 10Hz first and then 1Hz). During every rTMS treatment session (sham and active), participants received 1800 pulses targeted over the posterior, superior temporal gyrus. Magnetic pulses delivered at 1Hz occurred at the rate of 1 pulse per second for 30 minutes. Magnetic pulses delivered at 10Hz occurred in 72 trains, 2.5 seconds on and 30 seconds off, over 39 minutes. The TMS coil was located over the posterior, superior temporal gyrus by use of anatomical MRIs and the Brainsight® Frameless Stereotaxy System (Rouge Research). Coil position and location were tracked over the course of the study using the same system. The coil surface rested on the scalp and was positioned parallel to the cortical surface. The coil handle pointed toward the back and was aligned with the angle of the superior temporal gyrus.

### Electroencephalography (EEG)

Participants sat upright in a comfortable chair during EEG recording. The FPz, Cz, and Oz electrode placement locations were used to fit a TMS-compatible, 64- Ag/AgCl electrode cap (EasyCap, Brain Products GmbH, Gilching, Germany) to the participant’s head; secured by an elastic chin strap. Electrodes arranged in an extended 10-20 system were prepped with gentle scrubbing with a blunt-tip needle (MVAP Medical Supplies, Inc, Newbury Park, CA, USA) attached to a 10 mL Luer-Lok™ syringe (Becton, Dickinson & Company, Franklin Lakes, NJ, USA) or cotton-tipped applicator (Medline Industries, Mundelein, IL, USA), and an abrasive high-salt electrode paste (EasyCap, Brain Products GmbH, Herrsching, Germany). Electrodes were filled with electrode paste creating a “bridge” between the scalp and the electrode. Electrode impedances were kept below 10kΩ using the impedance monitor of the integrated Nicolet EEG recording system (Nicolet, NicVue, Natus Medical Inc., Pleasanton, CA, USA). Resting state EEG was recorded in a darkened room as participants remained still to minimize muscle artifact and as they directed attention to a focal point, a picture, to minimize eye movements. Alpha frequency and mean amplitude were examined in each epoch for consistency. Vigilance was monitored online for alpha slowing, drop-out, and the appearance of vertex sharp waves or sleep spindles. EEG signals were sampled at a rate of 1 kHz and filtered at 0.3-100Hz. The EEG was digitally recorded from the 61 non-reference/non-ground electrodes in ~ 1-minute cycles in both the eyes open and eyes closed condition, until at least 10 minutes were recorded. Twelve, 10-second epochs (120s total) of artifact free recordings were selected by a trained Neurologist (AS) from the recording in the eyes open and eyes closed condition as eye closure can affect power spectral levels, particularly in the alpha band [18].

Mean spectral power and magnitude of coherence in five frequency bands [delta (1–3.5Hz), theta (4–8Hz), alpha (9–12Hz), beta (14–28Hz) and gamma (30–50Hz)] were the primary EEG outcome measures. Exported EEG data were imported into MATLAB (The MathWorks, Natick, MA) using the EEGLAB toolbox. The EEG was transformed into reference free current density using the Hjorth transformation. Data were partitioned into one-second windows. The spectral power and coherence estimation followed the Welch periodogram approach. The power spectrum was computed for each band [delta (1–3.5Hz), theta (4–8Hz), alpha (9–12Hz), beta (14–28Hz), and gamma (30–50Hz)] as the Fourier transform of the autocorrelation function and then averaged across windows. Likewise, the cross-spectrum between two channels was computed for each window as the Fourier transform of the cross-correlation function and averaged. Power spectra were normalized by the total power across all frequencies. The coherence spectrum was the ratio of the square of the magnitude of the cross-spectrum to the product of the auto-spectra. Eigen mode decomposition was used to quantify the degree of connectivity between the channels. For each subject, coherence was calculated for each of the electrodes of focus (T7, T8, TP9, TP10, TP7, TP8, FT7, and FT8) with data from all other electrodes. Coherence values were averaged across all subjects for baseline, sham, 1Hz active, and 10Hz active.

### Analysis

Regarding tinnitus ratings, ANOVA for repeated measures was used to analyze ratings of tinnitus awareness, annoyance, and loudness. (As this study only concerned temporary changes in tinnitus that could be time-linked and correlated with changes in the EEG, data from the washout and follow up periods - the tinnitus questionnaires, and the TAP & TLM procedures - will be the subject of a second manuscript and based on a larger sample.) The statistical model examined effects of pre-post rTMS session, treatment day (1-3 for baseline, 1-4 for treatment), and treatment phase (sham, 1Hz and 10Hz rTMS). Differences between factor levels were estimated via Least Squares Means. The data and model residuals were inspected for normality. All analyses were done using SAS v9.4 (SAS Institute Inc., Cary, NC). The alpha level set for psychophysical data was <0.05.

Regarding analyses of the EEG, spectral power and coherence for each frequency band of the EEG was calculated for electrode location and each treatment phase and compared to the respective baseline value. Statistical significance of coherence was evaluated using a paired t-test by the 99% confidence limit. To address potential Type 1 error, *p* values <0.01 were considered significant. The ratios of spectral power and coherence for each frequency band, electrode, and treatment phase was also calculated by dividing the post-treatment value by the baseline value. These ratios were examined for the effects of the factors of side of stimulation (contra *vs.* ipsilateral to rTMS) and eyes open versus eyes closed via repeated measures ANOVA models. Each electrode ratio model included the factors: treatment phase (sham, 1Hz, 10Hz) side and eyes. [A 2-month follow-up was also recorded but not examined here because of missing data for several subjects.] The frequency of significance for the main effects of the factors side and eyes was then examined over all the electrodes for Spectral Power and for Coherence. Spearman correlations were used to associate changes in spectral power and coherence with changes in analogue ratings of tinnitus.

## Results

Participants were 19 Caucasian, non-Hispanic people (15 male, 4 female). Table 1 lists the baseline demographic and tinnitus characteristics. No serious, anticipated or unanticipated, study-related adverse events occurred. The anticipated study-related adverse events included headache and jaw pain. The unanticipated adverse events included missed study sessions due to non-study related illness or inclement weather. One non-study related, serious, unanticipated, adverse event occurred (a death).

### Change in VAR and LM ratings

A primary goal of the study was to examine the dissociations between one’s awareness of tinnitus and its psychophysical properties such as loudness, so each VAR and LM method included ratings of tinnitus loudness, annoyance and awareness in daily life. Only ratings for the ear contralateral to rTMS were used for data analysis due to a floor effect in the ipsilateral ratings. A floor effect occurred because some patients only have unilateral tinnitus.

#### Difference in tinnitus ratings before and after the rTMS sessions

There was no pre-post TMS session effect – no significant difference in the VAR or LM ratings, for tinnitus loudness, annoyance, or awareness, measured immediately before versus immediately after receiving rTMS. So, pre and post ratings were averaged in subsequent analyses. Nor were there any differences between the VAR or LM ratings. The two methods produced nearly identical results.

#### Difference in tinnitus ratings by day of treatment

**Figure 1.**
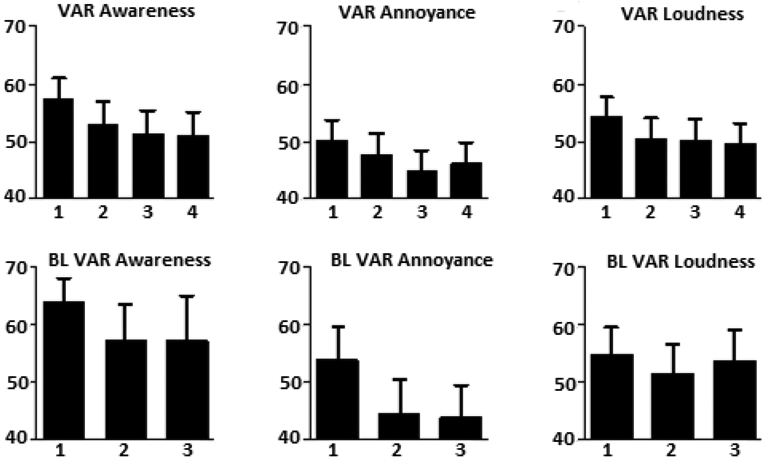
Bar graphs of the VAR ratings for tinnitus awareness, annoyance and loudness across each day of treatment (A) and each day of baseline (B: BL in the figure refers to baseline). LM ratings are not included in the graph. VARs of tinnitus drop across subsequent days of assessment during the baseline and treatment phases (sham and active). This effect represents a measurement confound called regression to the mean in tinnitus ratings.

Regarding days 1-3 of the baseline, VAR ratings, ratings made on day 2, for tinnitus loudness (*p*=0.0447), annoyance (*p*=0.0195), and awareness (*p*=0.0430), dropped significantly relative to ratings made on day one (Figure 1).

Regarding days 1-4 of the combined treatment phases (sham and active 1Hz and 10Hz rTMS), all of the tinnitus ratings except LM loudness and LM annoyance dropped significantly from the first to the second and from the first to third day of treatment (Table 2 and Figure 1). Ratings for both LM and VAR awareness also dropped from the first to the fourth day of treatment. LM loudness on the fourth day was significantly different from the first day rating.

**Figure 2.**
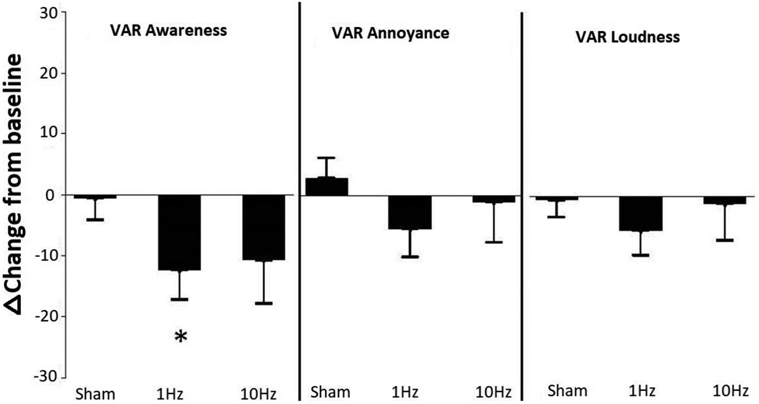
Change in VAR ratings by treatment phase. 1Hz rTMS reduced the VAR of tinnitus awareness from baseline. LM ratings, not shown, were nearly identical to the VARs.

For the active 1Hz rTMS treatment condition, both the LM and VAR awareness ratings were significantly different from baseline during the week of active 1Hz rTMS (VAR=12 point decrease, t=−2.47, df=100, *p*=0.0153; LM=12.5 point decrease, t=−2.41, df=49, *p*=0.0199). Ratings for annoyance and loudness were not different from baseline. No significant difference from baseline was observed for any ratings during sham and active 10Hz rTMS treatment (Figure 2).

#### Differences in EEG spectral power and coherence

Ten out of 24 electrodes showed a significant effect of side on the spectral power ratios while only 2 out of 24 showed significant effect of eyes. The two significant effects were just below the 0.05 cutoff level (*p*<0.0403 for Beta TP9 TP10 and *p*<0.0491 for Delta Gamma T7T8). Thus, side was retained as a factor in further analysis of the spectral power ratios while the eyes factor was eliminated.

Conversely, ten out of 24 electrodes showed a significant effect of eyes on the coherence ratios, while none showed a significant effect of side. Thus, eyes were retained as a factor in further analysis of the coherence ratios while the side factor was eliminated.

Table 3 summarizes the significant changes in spectral power and coherence from baseline for each frequency band of the EEG during sham and active rTMS. As noted above, eyes open and eyes closed conditions produced similar results for the ratios of spectral power. There were no changes in any frequency band of the EEG between baseline and sham rTMS. In contrast, spectral power increased significantly from baseline in the theta and alpha bands bilaterally and most strongly at the TP9/TP10 electrode locations for the active, 1Hz rTMS phase. Power also decreased from baseline in the beta and gamma frequency bands ipsilateral to active 1Hz rTMS. After 10Hz rTMS, spectral power increased in the delta, theta, and alpha frequency bands, most strongly at the TP9/TP10 electrode locations contralateral to rTMS. Spectral power decreased in the beta frequency band ipsilateral to active 10Hz rTMS and decreased in the gamma frequency band contralateral to rTMS at the TP9/TP10 electrode locations.

**Figure 3.**
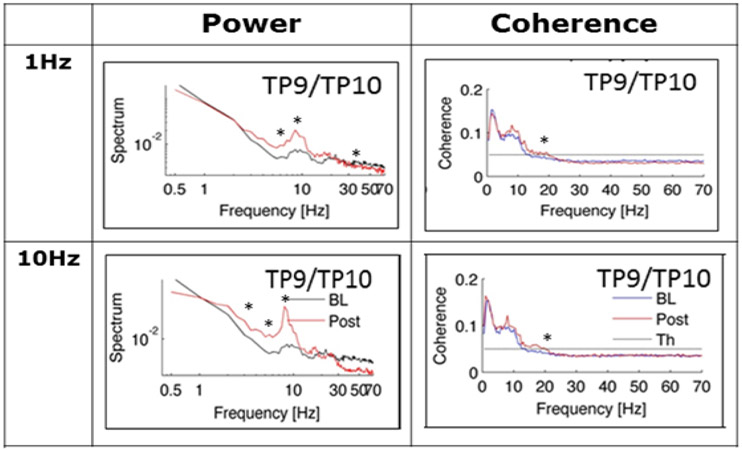
Examples of the significant changes in the EEG from baseline at the TP9/TP10 electrode site for 1Hz and 10Hz rTMS. After 1Hz rTMS, theta and alpha power increased, and gamma power decreased; beta coherence was increased from baseline. A similar pattern was observed for 10Hz rTMS except that delta power also increased and that these changes tended to be seen ipsilateral to rTMS.

EEG coherence increased bilaterally relative to baseline in the beta frequency band at the T7/T8 and TP9/TP10 electrode locations after active 1Hz rTMS. Coherence increased relative to baseline for the alpha and beta frequency bands, at the T7/T8, TP9/TP10 and FT7/FT8 electrode locations contralateral to rTMS after 10Hz active stimulation. Figure 3 shows a sample of the EEG power and coherence traces at baseline and post treatment week.

### Correlation between VAR and EEG power and coherence

For spectral power, the post/baseline ratio of spectral power was correlated with the post/baseline ratio of VAR ratings. Analyses were collapsed across the eyes open/closed condition because earlier comparisons showed little effect and preserved the ipsilateral/contralateral distinction. Similarly for the coherence correlations, the post/baseline ratio of coherence was correlated with the post/baseline ratio of VAR ratings; however, analyses collapsed across the ipsi/contralateral condition, which was not significant in ANOVA models for coherence ratios but preserved the eyes open/closed condition which was significant for about half of the comparisons. Significance was set to a p value <.01 to correct for multiple comparisons.

The main finding among correlations was that, for 1Hz rTMS, a decrease in tinnitus awareness was associated with an increase in beta (FT7/8, r=−0.51, *p*<0.001) and delta coherence (T7/8, r=−0.50, *p*<0.001). Additionally, a decrease in tinnitus annoyance was associated with increased alpha (TP9/10, r=−0.51, *p*<0.001) and beta coherence (FT7/8, r=−0.51, *p*<0.002); however, unlike ratings of awareness, those for annoyance did not change significantly from baseline in the 1Hz rTMS treatment phase.

Other correlations that achieved significance were between frequency bands of the EEG and VAR ratings that did not change significantly from baseline as a result of rTMS treatment. For example, for 10Hz rTMS, VAR loudness increased significantly with increases in both theta and beta coherence, (T7/T8, r=0.51 & 0.49, *p*<0.003, respectively) but loudness did not change significantly from baseline as a result of either 1 or 10Hz rTMS. So, these correlations were not relevant to a significant change in tinnitus perception following treatment. All of the correlations between VAR ratings and spectral power were of this type and can be summarized as follows: for the electrodes located contralateral to 1Hz rTMS, tinnitus loudness and annoyance was negatively correlated with delta, theta, and alpha power and positively correlated with beta and gamma power. The same relationships were observed for theta, alpha and gamma power during sham stimulation. In general, results for 10Hz rTMS were opposite those for 1Hz rTMS. First, significant correlations were observed ipsilateral rather than contralateral to rTMS. Second, the correlations were in an opposite direction. Loudness and annoyance were positively correlated with theta and alpha power and negatively correlated with gamma power.

### Exploratory analysis of the relationship between treatment response and change in the EEG

**Figure 4.**
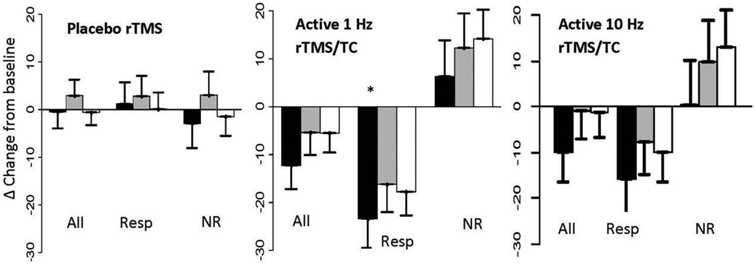
Change in tinnitus ratings in from baseline after placebo and active 1Hz & 10Hz rTMS/TC. Black bars refer to tinnitus awareness, grey bars refer to annoyance, and white bars refer to loudness. Data are shown in clusters for all subjects combined (All), for responders (Resp) and non-responders (NR). In contrast to placebo rTMS, which does not change tinnitus, active 1Hz rTMS/TC lead to greater change in tinnitus awareness than loudness or annoyance in positive responders and to an opposite pattern in negative responders. 10Hz had a similar but n.s. effect.

rTMS treatment studies of tinnitus have recognized that some patients respond well to treatment (i.e., treatment responders) while others do not (non-responder). So, participants in the current study were classified as a treatment responder if any of their VAR or LM ratings of tinnitus fell by more than 30% from baseline during treatment. Eleven subjects (58%) met criteria for a treatment responder and 8 patients (42%) met criteria for a non-responder. Whereas the VARs of awareness, annoyance, and loudness were not different between treatment responders and non-responders at baseline or following sham rTMS; the ratings of awareness for treatment responders decreased from baseline during active 1Hz rTMS (df=58, t=−2.25, *p*<0.02) (Figure 4). In contrast, the ratings for non-responders tended to increase from baseline but these changes were not significant.

EEG data were then analyzed to explore the differences between responders and non-responders to 1Hz rTMS versus sham rTMS. Table 4 shows differences in power and coherence between all subjects receiving sham and active 1Hz rTMS versus the responders and non-responders to 1Hz rTMS.

Whereas for all subjects a significant increase in beta coherence and in theta-beta spectral power, and a decrease in gamma power, is observed following active 1Hz rTMS relative to sham stimulation; the strength of the response was increased contralaterally in treatment responders. In contrast, findings for non-responders were similar to sham rTMS.

Ratios of spectral power and coherence relative to baseline were correlated with the ratios of tinnitus awareness, annoyance and loudness relative to baseline using Spearman correlation. Relative to the larger sample, some differences were observed when the data were split by the treatment responder/non-responder category. In treatment responders, a decrease in tinnitus awareness correlated with a decrease in gamma power (r=0.56, p<0.006) and with an increase in alpha and beta coherence (r=−71, p<0.0002, r=−0.53, *p*<0.01, respectively). In non-responders, significant correlations were between frequency bands of the EEG and VAR ratings that did not change significantly from baseline.

## Discussion

According to the TCD model of tinnitus [10,11], improvement in tinnitus following active rTMS should be associated with decreased spectral power and coherence for low-frequency EEG bands (delta-theta) and an increase power and coherence in the higher-frequency bands (alpha-beta). In fact, active 1Hz rTMS significantly decreased tinnitus awareness from baseline, a pattern consistent with habituation, and a decrease in tinnitus awareness correlated most consistenly with an increase in beta coherence and a decrease in gamma power. This pattern was amplified in treatment responders. Changes in spectral power from baseline were also associated with 1Hz rTMS. Alpha power increased and gamma power decreased, as predicted by the TCD model, but power in the theta range increased rather than decreased. In general, changes in spectral power did not correlate with changes in tinnitus awareness. They correlated instead with change in tinnitus loudness and annoyance which themselves did not change significantly from baseline after active rTMS at any frequency. Whereas 10Hz rTMS induced similar trends in tinnitus ratings and similar changes in the EEG from baseline; they were less robust than 1Hz rTMS. Sham rTMS did not alter tinnitus ratings or the EEG relative to baseline. Finally, the EEGs of nonresponders to active rTMS were unchanged from baseline. It is concluded, therefore, that a positive response to rTMS for tinnitus is associated with an rTMS-induced change in beta coherence of the EEG and a decrease in gamma power. Beta coherence may reflect a “top-down” modulation of the EEG that promotes habituation to tinnitus. The remainder of the discussion provides the context for these interpretations.

### Convergence with other studies

We know of only one other study that used EEG to examine changes in spectral power before and after rTMS in patients with tinnitus [19]. The results of this study are consistent with our own in showing sensitivity of EEG to rTMS-induce change in neuroplasticity; however, the design is more of an experimental rather than a treatment protocol. So, the results are not directly comparable. Stimulation consisted of 200 pulses at 1Hz and 60% of the MSO, delivered one time in each of four locations – left and right temporal and prefrontal cortex. Subjects received stimulation at three locations in the same hemisphere during a single test session. EEG was collected four times per session – before, in between and after rTMS. Whereas immediate changes in spectral power, including a decrease in gamma power, were observed after rTMS to the right frontal location; the brief rTMS exposures could not produce a reliable change in visual analogue measures of tinnitus loudness. So, immediate change in the EEG could not be correlated with an immediate change in analogue ratings of tinnitus. Our study also found that a single rTMS session is not sufficient to produce reliable change in analogue ratings of tinnitus.

A second study compared 1Hz rTMS over temporal cortex to an EEG source analysis guided approach [20]. EEG was not the outcome measure but rather a means of guiding placement of the rTMS coil. Interestingly, following 10 rTMS treatments, the source analysis approach was superior at reducing tinnitus relative to placing the coil over temporal cortex. The source site most associated with a positive response to rTMS was the right frontal lobe. Additionally, 10 days of rTMS treatment produced significant changes in both the Tinnitus Handicap Inventory and analogue ratings of tinnitus loudness. Wang’s study reveals yet another way in which TMS and EEG can be combined in tinnitus studies – to guide coil placement as well as to assess neurophysiological responses to treatment – and it suggests that a greater number of rTMS treatments may be necessary to change tinnitus loudness.

### How rTMS may change tinnitus perception

Over four days, 1Hz rTMS had a significant effect on tinnitus awareness but not loudness or annoyance. This outcome could be due, in part, to delivering only four days of treatment, which was intended in our study to produce a temporary change in tinnitus so that 1Hz and 10Hz rTMS could be compared within subjects. Four days of treatment may not be sufficient to change annoyance and loudness. Alternatively, the outcome could be due to low statistical power owing to a small sample size. Follow-up power calculations showed that tinnitus ratings have different effect sizes to discriminate between sham and active rTMS. For example, based on data from the current study, 40 subjects would be sufficient to observe significant changes at a *p*<0.05 level in tinnitus annoyance and 60 subjects would be sufficient to observe changes in tinnitus loudness. So, 1Hz rTMS might also reduce tinnitus annoyance and loudness but a larger sample of subjects is needed to test this hypothesis. Likewise, while neither sham nor active 10Hz rTMS decreased any analogue ratings of tinnitus significantly from baseline, power calculations indicated that for 10Hz rTMS (but not sham) changes in tinnitus awareness could reach significance at a *p*<0.05 level with 60 subjects.

Even with these cautions in mind, however, the changes in analogue ratings of tinnitus appear to have occurred for two main reasons. First, all tinnitus ratings (even those obtained over three baseline observations) dropped from the first to the second and third days of assessment. The most coherent explanation for a drop like this is regression to the mean – a tendency for extreme ratings to drop with successive measurement. Regression to the mean is a measurement confound that can mimic a treatment effect, particularly a placebo effect, when the initial ratings are extremely negative [21]. In fact, in the current study, the highest tinnitus ratings were consistently observed on the first days of the baseline and treatment periods and they dropped thereafter. So, in general, regression to the mean mimicked improvement in this study. It is likely that regression to the mean is widespread in studies of tinnitus, but we do not know of any rTMS studies that have specifically measured regression to the mean. So, it is necessary in future studies to have an extended baseline and to measure tinnitus repeatedly during treatment and follow-up. Only in this way can regression to the mean be included in the analyses. Fortunately, our finding implies that regression to the mean can be directly assessed in clinical trials to determine its influence.

The second reason for how analogue ratings changed was that active rTMS decreases tinnitus awareness more than loudness or annoyance. Active, 1Hz rTMS decreased tinnitus awareness significantly from baseline even when accounting for regression to the mean. Active 10Hz rTMS was less effective than 1Hz rTMS and sham rTMS did not have an effect that could not be explained by regression to the mean. This pattern of change suggests that 1Hz rTMS promotes habituation to tinnitus.

The prefrontal cortex gates sensory information from the thalamus via cortico-limbic-reticular-cortical circuits [8]. Activation of the prefrontal cortex excites the reticular nucleus which sends GABAergic inhibitory fibers to sensory relay nuclei. Ablative lesions of both the prefrontal and parietal cortex can disrupt habituation in opposite ways. Discrete lesions of the prefrontal cortex amplify auditory evoked response potentials generated in the primary auditory cortex, whereas lesions of the temporo-parietal cortex reduce the amplitude of auditory evoked potentials [22]. Similarly, discrete lesions of the premotor and prefrontal cortex can retard habituation to visual stimuli, while lesions of the inferior parietal cortex can accelerate habituation [23]. Given the role of the frontal cortex in sensory gating, it is possible that rTMS delivered over temporal cortex promotes habituation to tinnitus by activating/engaging regions of the prefrontal cortex that habituate attention to redundant sensory information. This interpretation is consistent with findings in other studies that delivered rTMS to both temporal and prefrontal cortex and observed augmented effects [24,25]. It might be how increased beta coherence following temporal lobe rTMS correlates with a decrease tinnitus awareness; by increasing neural synchrony with brain regions like the frontal lobe.

Another way in which rTMS changes ratings of tinnitus is cumulatively, occurring over 3-4 days, or possibly longer, rather than immediately following stimulation. The lack of an immediate effect is interpreted to mean that the rTMS effect on tinnitus is not frequency dependent, i.e., it does not depend on an immediate change in cortical activation induced by different frequencies of rTMS. This may be how previous studies showed that courses of 1Hz, 10Hz and 25Hz rTMS were all effective at improving tinnitus. It is possible that the degree of tinnitus improvement is related to the cumulative number of pulses delivered rather than the pattern at which these pulses are delivered (in this study, an identical number of 1800 pulses were delivered per session either at 1Hz or 10Hz). The fact that the effect of rTMS on tinnitus has to be conditioned over time implies that rTMS either induces a return to homeostasis in neural activity of the cell assemblies in temporal cortex following repeated, simultaneous activation or that it induces a change in the activity of brain regions or networks that are functionally connected to temporal cortex and influence tinnitus awareness or both possibilities.

Changes in the EEG may offer clues about the mechanisms by which rTMS has a conditioned effect. First, it is important to point out that sham stimulation had little to no effect on EEG spectral power or coherence from baseline. Second, changes in the EEG were only present in participants who also reported improvement in tinnitus. The EEGs of non-responders were essentially the same as those for sham rTMS (i.e., no conditioned effect). In treatment responders, however, significant increases in delta, theta, and alpha spectral power and decreases in gamma power were evident after both 1Hz and 10Hz rTMS. These changes tended to occur bilaterally for 1Hz rTMS and contralaterally for 10Hz rTMS, which might explain how 1Hz rTMS had a more robust effect on tinnitus ratings.

It is also important to note, that changes in spectral power, other than gamma power, were not associated with the apparent therapeutic effect of rTMS on tinnitus - to reduce tinnitus awareness. Nearly all of the significant correlations between spectral power and ratings of tinnitus loudness and annoyance were in EEG frequency bands and at electrode locations that did not change significantly from baseline after active rTMS. Instead, they were already present at baseline. So, either changes in spectral power are not a good biomarker of beneficial change in tinnitus, or our sample size was too small to detect them, or changes in spectral power operate in a more complicated way with other changes like those for coherence.

A much clearer relationship emerged between rTMS induced change in tinnitus awareness and EEG coherence - a measure of dynamic functional interactions between electrode signals [15]. A 4-day course of 1Hz rTMS promoted habituation to tinnitus and the biomarker of change associated with this effect was an increase in beta coherence. Distinguishing between responders and non-responders not only strengthens this finding, but it also showed that the EEG did not change from baseline in participants whose tinnitus did not change from baseline. Beta coherence may play a role in the cross-frequency coupling that promotes communication between different brain regions like temporal and frontal cortex [26,27]. Other networks might also be involved. Increased coherence may reflect greater functional cooperation between networks. Unfortunately, a limitation of our approach is that we cannot determine the source of the EEG signals.

In a study that used depth electrode recording from two auditory cortical regions in order to study bottom-up and top-down propagation of sensory processing (listening to sentences); bottom-up processing dominated in the gamma range whereas top-down processing dominated in the delta-beta bands. These data suggested that the power of gamma activity in one auditory area is modulated by the phase of delta-beta activity in another auditory area [27]. Our data showed that beta coherence was increased and uniquely correlated with a reduction in tinnitus awareness and that gamma power decreased. This relationship suggests that phase coupling may have exerted a modulatory, top down, influence on tinnitus perception in which increased beta coherence may modulate gamma power. Beta activity may be particularly able to synchronize over long distances in the brain [28] and be a vehicle for cooperation between higher order association areas mediating attention or emotion [26].

## Conclusion

Active 1Hz rTMS promotes temporary habituation to tinnitus – a decrease in daily awareness but not focused ratings of loudness or annoyance. This effect was cumulative rather than immediate and it correlated with an increase in beta coherence of the resting state EEG. Participants whose tinnitus did not improve after rTMS did not show changes in the EEG. Beta coherence could be a potential biomarker of the rTMS effect on tinnitus. Regression to the mean emerged as an important threat to internal validity that could explain placebo effects which are common in rTMS studies of tinnitus. Regression can be accounted for in clinical trials by using extended baselines and repeated assessments of tinnitus during treatment and follow-up.

## Figures and Tables

**Table 1.** Subject baseline characteristics

Subject Characteristics (N=19)			
**Demographics**	Age	median (range)	54 (23-70)
	Sex	Male	15 (79%)
		Female	4(21%)
	Race and Ethnicity	White	19 (100%)
		Non-Hispanic	19 (100%)
**Tinnitus Characteristics**	Tinnitus Duration	Less than 1 year	1 (5%)
		Between 1 and 2 years	2 (11%)
		Between 2 and 3 years	5 (26%)
		Between 3 and 4 years	2 (11%)
		More than 5 years	9 (47%)
	Change in tinnitus loudness before study	Much quieter	0 (0)
		Quieter	0 (0)
		No different	6 (32%)
		Louder	9 (47%)
		Much louder	4 (21%)
	Average percent time awake annoyed by tinnitus	Average	28%
	Average percent time awake aware of tinnitus	Average	59%
	Tinnitus effect on concentration	Never	1 (5%)
		Rarely	5 (26%)
		Sometimes	8 (42%)
		Frequently	3 (16%)
		All the time	2 (11%)
	Tinnitus effect on falling asleep	Never	4 (21%)
		Rarely	5 (26%)
		Sometimes	8 (42%)
		Frequently	2 (11%)
		All the time	0 (0)
**Tinnitus Matching Loudness**	Hearing Level in dB (HL)	average	42.7
	Sensation Level in dB (SL)	average	13.72
	Average Baseline Tinnitus Comorbidities Scores	Beck’s Depression Inventory (BDI)	5.58
		State Trait Anxiety Inventory (STAI)	25.57
	Average Baseline Tinnitus Questionnaire Scores	Tinnitus Handicap Inventory (THI)	30.16
		Tinnitus Handicap Questionnaire (THQ)	35.82%
	THQ Factor Scores	Social, Emotional, Behavioral	29.01%
		Hearing	32.45%
		Outlook	47.11%

**Table 2.** *P*-values pertaining to significant drops in tinnitus ratings from day 1 through day four of treatment phases. The sham, 1Hz and 10Hz conditions were combined for these comparisons. Ns refers to a non-significant *p*-value (<0.05 for the psychophysical data).

Rating type	Day 2 *vs*. Day 1	Day 3 *vs*. Day 1	Day 4 *vs*. Day 1
**LM - Loudness**	**ns**	**ns**	*p*=0.0329
**LM - Annoyance**	**ns**	**ns**	**ns**
**LM - Awareness**	*p*=0.0483	*p*=0.00717	*p*=0.0289
**VAR - Loudness**	*p*=0.0413	*p*=0.0312	**ns**
**VAR - Annoyance**	*p*=0.0386	*p*=0.0052	**ns**
**VAR - Awareness**	*p*=0.0197	*p*=0.0007	*p*=0.0297

**Table 3.** Changes in spectral power and coherence from baseline for each frequency band of the EEG during sham and active rTMS.

Location Side		Contralateral Power/Coherence								Ipsilateral Power/Coherence							
Electrodes		T7/T8		TP9/TP10		TP7/TP8		FT7/FT8		T7/T8		TP9/TP10		TP7/TP8		FT7/FT8	
Eyes open/closed		O	C	O	C	O	C	O	C	O	C	O	C	O	C	O	C
**Sham**	delta																
	theta																
	alpha							↑									
	beta																
	gamma																
**1Hz**	delta																
	theta			↑	↑							↑	↑	↑			
	alpha			↑	↑				↑			↑	↑	↑			
	beta		↑	↑	↑					↑	↑	↑	↑	↑			↑
	gamma											↓	↓				
**10Hz**	delta			↑	↑												
	theta			↑	↑												
	alpha	↑		↑	↑			↑		↑							
	beta			↑	↑					↑	↑						
	gamma				↓												

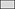
 Light box indicates a change in Spectral Power from baseline; ↑=increase, ↓=decrease

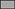
 Dark box indicates a change in Coherence from baseline

**Table 4.** The top box shows results for all subjects receiving sham rTMS (all sham) and the non-responders to active 1Hz rTMS (1Hz NR). The middle box shows results for all subject receiving active 1Hz rTMS. The bottom box shows results for only treatment responders to active 1Hz rTMS. Whereas non-responders produced results similar to sham rTMS; changes in the EEG in response to active 1Hz rTMS were amplified in among treatment responders

Location Side		Contralateral Power/Coherence								Ipsilateral Power/Coherence							
Electrodes		T7/T8		TP9/TP10		TP7/TP8		FT7/FT8		T7/T8		TP9/TP10		TP7/TP8		FT7/FT8	
Eyes open/closed		O	C	O	C	O	C	O	C	O	C	O	C	O	C	O	C
**All Sham/1Hz NR**	delta																
	theta																
	alpha							↑									
	beta																
	gamma																
**All 1Hz**	delta																
	theta			↑	↑							↑	↑	↑			
	alpha			↑	↑				↑			↑	↑	↑			
	beta		↑	↑	↑					↑	↑	↑	↑	↑			↑
	gamma											↓	↓				
**TR 1Hz**	delta		↑														
	theta	↑	↑	↑	↑	↑		↑				↑	↑	↑			
	alpha	↑	↑	↑	↑			↑				↑	↑	↑			
	beta			↓	↑						↑		↑				↑
	gamma		↓		↓							↓	↓				

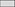
 Light box indicates a change in Spectral Power from baseline; ↑=increase, ↓=decrease

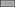
 Dark box indicates a change in Coherence from baseline

All: All subjects combined, 1Hz: Active 1Hz rTMS/TC, NR: non-responder, TR: treatment responder
